# Fashion and Common Sense

**Published:** 1888-05

**Authors:** 


### FASHION AND COMMON SENSE.

New York City is the Mecca to which our Jewish ladies who live beyond its borders, look with longing eyes and hopeful hearts.

A pilgrimage to its dazzling scenes is the height of their ambition, and its realization a red letter page in their life's history, painted in fervid carmine colors and brilliant with gold leaf scintillations.

The delightful shopping, the exquisite downtown lunches, the ravishing bonnets, the enchanting matinees, the sweet toilets, the wonderful *metseeas*, the charming concerts, the enjoyable drives, the witching opera bouffes, the elegant gentlemen, the romantic sail up the Hudson, the merry midsummer night's festival, the gorgeous opera, the trip to Coney Island, the fashionable surf bathing at Long Branch, the moonlight excursion, the balls, receptions, etc., all form a varied kaleidoscope of delightful reminiscences, which the mental eye never tires of dwelling upon or the willing tongue of eloquently illustrating.

And the average New York girl somehow conceives the idea that all beyond the boundaries of Gotham is a dreary waste and howling wilderness, peopled with women whose conceptions of society are as crude as their dresses are unfashionable, and men who are as uncouth in their manners as they are untutored in the amenities of civilized life.

Mr. Leon Goldschmidt, of Tackleville, Va., was introduced a few years ago by a friend of the family, and we were told *entre nous*, that he had matrimonial inclinations. He came to the city twice a year to purchase goods, during which time he visited the house and paid marked attention to Hannah. He was an affable gentleman, an agreeable conversationalist and very pleasant in his manners.

He gave pa references as to his family connections, and asked permission to correspond with Hannah, to which, after examining his record at the mercantile agencies and finding out all about him, pa assented.

He ultimately proposed to Hannah, and to the astonishment of everybody except Hannah and mamma, was refused ; simply because she did not want to live in the country Pa expostulated with her in vain, told her that in a few years he would doubtless move to New York, that he would not ask her to marry him unless she loved him, and if she did, she ought to be willing to go with him to Hong Kong, if he desired, but it was all useless. Hannah would not leave New York, and the idea of living in Tackleville, even if it was near the Hot Springs, was preposterous, and Mamma said she never would consent that Hannah should be buried alive in a small country town, and what ma says is law in our house.

Hannah will be thirty-five next Chanukah, and Mr. Goldschmidt is a prosperous merchant on Broadway. He resides in sumptuous style on Madison avenue, and his wife is one of the leaders of fashionabl society. Hannah will accept any offer now, and would be willing to go even to Yerusholayim.

Our young married ladies lead such a supposed life of gayety that our daughters imagine that married life without Kaffee Klatsches, theatre parties and even poker playing, would scarce be worth the living.

They forget that real, true enjoyment of life in all that the word implies, is almost an impossibility in a large crowded city. That real married happiness can best be obtained in the attractive domestic circle, and that all the fancied enjoyments of fashionable life are more than counterbalanced by the poker parties, club meetings, lodge suppers and kegel tournaments toward which the equally fashionable husband gravitates. There is less social merriment in the country, less style, it is true, but less expense. In the country you have a home and you live in it ; in the city you are more in other people's houses than in your own.

How many Jewish maids would, to-day, have been devoted wives and happy mothers had they been less fashionable and more sensible.—*Hebrew Standard.*

